# ENDOSCOPIC ABLATION COMBINED WITH FUNDOPLICATION PLUS ACID SUPPRESSION-DUODENAL DIVERSION PROCEDURE FOR LONG SEGMENT BARRETT´S ESOPHAGUS: EARLY AND LONG-TERM OUTCOME

**DOI:** 10.1590/0102-672020230042e1760

**Published:** 2023-09-18

**Authors:** Italo Braghetto, Héctor Valladares, Enrique Lanzarini, Maher Musleh, Attila Csendes, Manuel Figueroa-Giralt, Owen Korn

**Affiliations:** 1 Universidad de Chile, Faculty of Medicine, Hospital “Dr. José J. Aguirre”, Department of Surgery – Santiago, Chile.

**Keywords:** Barrett Esophagus, Endoscopy, Radiofrequency Ablation, Argon Plasma Coagulation, Fundoplication, Gastrectomy, Esôfago de Barrett, Endoscopia, Ablação por Radiofrequência, Coagulação com Plasma de Argônio, Fundoplicatura, Gastrectomia

## Abstract

**BACKGROUND:**

The addition of endoscopic ablative therapy plus proton pump inhibitors or fundoplication is postulated for the treatment of patients with long-segment Barrett´s esophagus (LSBE); however, it does not avoid acid and bile reflux in these patients. Fundoplication with distal gastrectomy and Roux-en-Y gastrojejunostomy is proposed as an acid suppression-duodenal diversion procedure demonstrating excellent results at long-term follow-up. There are no reports on therapeutic strategy with this combination.

**AIMS:**

To determine the early and long-term results observed in LSBE patients with or without low-grade dysplasia who underwent the acid suppression-duodenal diversion procedure combined with endoscopic therapy.

**METHODS:**

Prospective study including patients with endoscopic LSBE using the Prague classification for circumferential and maximal lengths and confirmed by histological study. Patients were submitted to argon plasma coagulation (21) or radiofrequency ablation (31). After receiving treatment, they were monitored at early and late follow-up (5–12 years) with endoscopic and histologic evaluation.

**RESULTS:**

Few complications (ulcers or strictures) were observed after the procedure. Re-treatment was required in both groups of patients. The reduction in length of metaplastic epithelium was significantly better after radiofrequency ablation compared to argon plasma coagulation (10.95 vs 21.15 mms for circumferential length; and 30.96 vs 44.41 mms for maximal length). Intestinal metaplasia disappeared in a high percentage of patients, and histological long-term results were quite similar in both groups.

**CONCLUSIONS:**

Endoscopic procedures combined with fundoplication plus acid suppression with duodenal diversion technique to eliminate metaplastic epithelium of distal esophagus could be considered a good alternative option for LSBE treatment.

## RESUMO

**RACIONAL:**

A adição de terapia ablativa endoscópica associado a inibidores da bomba de prótons ou fundoplicatura tem sido postulada para o tratamento de pacientes com esôfago de Barrett de segmento longo (EBSL), no entanto, essa conduta não evita o refluxo ácido/biliar nesses pacientes. A fundoplicatura com gastrectomia distal e gastrojejunostomia em Y de Roux (FGD-Y) foi proposta como procedimento de supressão de ácido, demonstrando excelentes resultados no seguimento a longo prazo. Não há relatos na literature com a combinação dessa estratégia terapêutica.

**OBJETIVOS:**

Determinar os resultados precoces e a longo prazo observados em pacientes com EBSL com ou sem dysplasia de baixo grau, submetidos a FGD-Y, combinado com terapia endoscópica.

**MÉTODOS:**

Estudo prospectivo incluindo pacientes com EBSL, empregando a classificação de Praga, sendo o comprimento circunferencial (C) e máximo (M) e confirmado por estudo histológico. Os pacientes foram submetidos à coagulação com plasma de argônio (CPA, 21 pacientes) ou ablação por radiofrequência (ARF, 31 pacientes). Após o tratamento, eles foram seguidos precoce e tardiamente (5–12 anos), mediante avaliação endoscópica e histológica.

**RESULTADOS:**

Foram observadas poucas complicações após o procedimento (úlcera ou estenose). Re-tratamento foi necessário em ambos os grupos de pacientes. A redução do comprimento do epitélio metaplásico foi significativamente melhor após ARF em comparação com CPA (10,95 versus 21,15 mm para C e 30,96 versus 44,41 mm para M). A metaplasia intestinal desapareceu em elevada porcentagem de pacientes, e os resultados histológicos a longo prazo foram bastante semelhantes em ambos os grupos.

**CONCLUSÕES:**

Procedimentos endoscópicos combinados com fundoplicatura e gastrectomia distal e gastrojejunostomia em Y de Roux, para eliminar o epitélio metaplásico do esôfago distal podem ser considerados uma boa opção alternativa para o tratamento da EBSL.

## INTRODUCTION

Chronic gastroesophageal reflux disease (GERD) is associated with the metaplastic transformation of normal squamous epithelium to premalignant specialized intestinal metaplasia (IM), frequently within the long segment of Barrett´s esophagus (LSBE), which may progress to low-grade dysplasia (LGD), high-grade dysplasia (HGD), or even esophageal adenocarcinoma (EAC). The results of treatment with proton pump inhibitors (PPIs) or surgery with fundoplication alone are intensively discussed among gastroenterologists and surgeons^11^. Medical treatment fails to control bile reflux, and the reflux of acid and bile after an ineffective antireflux barrier plays a fundamental role in the genesis and progression of IM and dysplasia^4,19,29^. Endoscopic techniques for the eradication of IM and dysplasia are proposed, including endoscopic submucosal dissection (ESD), endoscopic radiofrequency ablation (RFA), argon plasma coagulation (APC), photodynamic therapy, and cryotherapy^18,26,28,41,44,45^. Among the ablative modalities, RFA is the most employed^34^.

A large volume of published data consistently documented high rates of complete eradication of IM and dysplasia, reduced risk of EAC, and low rates of complications, thus establishing RFA as the preferred modality^4,49^. While this procedure can eradicate either IM or LGD, recurrence can be observed over time once acid and bile reflux are not permanently eliminated with medical treatment.

The combination of fundoplication with endoscopic therapy is postulated as a valid option, showing good initial and long-term results^27^. However, laparoscopic fundoplication in LSBE patients fails to create an effective and permanent antireflux barrier, in almost 30% of cases^6,14^. Fundoplication combined with highly selective vagotomy is associated with high rate of recurrence of Barrett's esophagus (BE) and its complications^17^.

Therefore, we performed the fundoplication with distal gastrectomy and Roux-en-Y gastrojejunostomy in LSBE patients to avoid acid and bile reflux, called acid suppression, and obtain an early and safe eradication with diversion procedure, known as acid suppression-duodenal diversion (AS-DD). With this procedure, regression of IM to cardiac mucosa was observed in 38% and LGD to IM in 80% of cases, at very long follow-up^14,17,35^. Despite these excellent results, we propose to add ablation in patients with LSBE C5M5 (Prague classification^3^) or more to obtain early and permanent elimination of IM or LGD, considering the risk of progression at an incidence rate, for any dysplasia, of 1.4 cases/100 person-years and HGD/EAC of 0.9/100 person-years^31,39^. Therefore, in this way, we can prevent disease progression.

Up to date there are no reports with the combination of this procedure with endoscopic therapy.

The objective of this study was to determine the early and long-term results observed in LSBE patients (with or without LGD) who underwent AS-DD procedure combined with endoscopic therapy.

## METHODS

### Patients

This is a prospective study including patients with LSBE confirmed by endoscopic and histological study that demonstrated the presence of IM and/or LGD (inclusion criteria). In order to have a very clean universe of participants, exclusion criteria considered patients with short-segment BE (<3 cm), hiatal hernia >4 cm, previous esophago-gastric surgery, obesity with BMI>32, adenocarcinoma of the esophagus, and those HGD submitted to ESD. The admitted group consisted of 52 patients, 28 men with a mean age of 55.9 years (range 41–71) and 24 women with a mean age of 54.1 years (range 35–71). All of them had repeated endoscopic and histologic diagnoses of LSBE C5M5 or more for at least three years before their inclusion in this study. They underwent two endoscopic procedures in two different periods depending on the endoscopic therapy availability. A group of 21 patients received APC between March 2010 and December 2012, and another group of 31 patients underwent RFA from March 2012 to December 2019. Table 1 shows the demographic characteristics of the patients and BE extension of each group. They underwent endoscopic procedure between three and six months after AS-DD technique.

**Table 1:** Demographic characteristics of patients submitted to argon plasma coagulation or radiofrequency ablation for the treatment of Barrett's esophagus.

			APC n=21	RFA n=31
Age (range)			55.9 (45–71)	54.1 (35–71)
Sex (n)				
	Male		12	16
	Female		9	15
Endoscopy (Prague classification^3^)				
	C (mm) (mean±SD)		30.47±8.05	47.30±9.6
	M (mm) (mean±SD)		54.76±9.81	71.33±18.1
		C3-5 M5	9	8
		C5-7 M7	7	17
		C>7 M7	5	6
Histology				
	Intestinal metaplasia		20 (95.2%)	25 (80.6%)
	Intestinal metaplasia+LGD		1 (4.8%)	6 (19.4%)

APC: argon plasma coagulation; RFA: radiofrequency ablation; C: circumferential length; M: maximal length; SD: standard deviation; LGD: low grade dysplasia.

The present study was performed in line with the Helsinki Declaration principles. Approval was granted by the Ethics Committee of our hospital and written consent was applied to patients before the indication of the surgical procedure. All patients gave their written informed consent to be included in this study.

### Surgical technique

Nissen fundoplication plus selective vagotomy, distal gastrectomy with Roux-en-Y gastrojejunostomy with an alimentary limb of at least 80 cm was performed according to a previously described AS-DD plus fundoplication technique^5,14,35^.

Postoperatively, upper endoscopy was performed to determine the onset and characteristics of the Barrett's epithelium according to the Prague classification^3^. Measurements were performed to determine the circumferential and maximal lengths of the metaplastic epithelium, from the gastroesophageal junction towards the proximal.

### Histologic analysis

According to the current guidelines^7,9,14,15,16^, circumferential and staggered biopsies 4-quadrant with 1 cm each were removed from the proximal border of the Barrett´s epithelium until close to the gastric esophagogastric junction (EGJ)^47^. The number of biopsies for each patient ranged from 18 to 56 depending on the follow-up period. Cardiac mucosa was described as the presence of mucus-secreting columnar cells (carditis). Specialized columnar epithelium was characterized by the presence of IM with well-defined goblet cells. LGD was defined as the presence of nuclear atypia involving the mucosal surface, nuclear stratification in the crypt base, and preserved architecture. HGD corresponded to the presence of marked nuclear atypia, distorted crypt architecture, and nuclear stratification extending to luminal surface.

### Equipment and procedure

**Figure 1:**
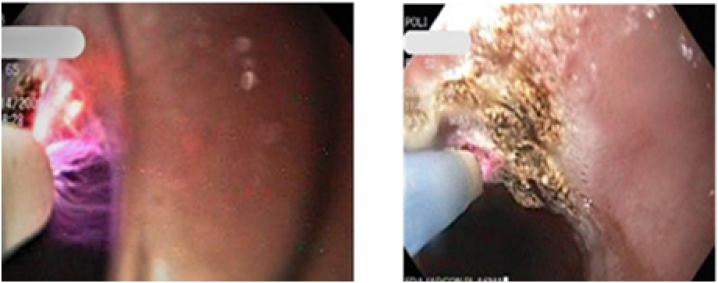
Endoscopic treatment with argon plasma coagulation.

For the APC, it was used an electrosurgical unit with Argon module EMED™ ES 350-100-008 (EC manufacturer, Ryzowa, Poland) with a reusable flexible probe, GIT, 2.3 mm diameter, 2,2 m length. The argon beam was applied to produce the electrical coagulation and complete removal of metaplastic surface endoscopically visualized (Figure 1).

As for RFA, gastrointestinal ablation system Barrx™ RFA energy generator (Medtronic Corporation, BARRX Medical, Sunnyvale, California) was used. After determining the length and characteristics of the ablation area, a sizing balloon catheter was initially introduced to determine the diameter of the ablation catheter to be used. For the treatment of wide surfaces of Barrett's epithelium, the circumferential flex ablation (360º express) balloon catheter (3 cm length, 31 mm diameter) was initially introduced, administering a brief burst of <1s of thermal energy applied circularly to the esophageal wall. In the last patients, we used the self-sizing balloon catheter.

**Figure 2:**
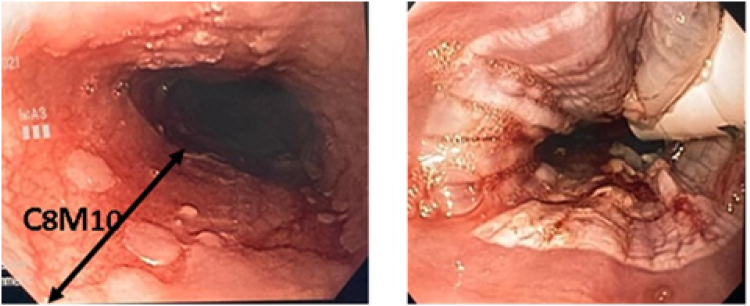
Endoscopic visualization before and after radiofrequency ablation for long segment Barrett´s esophagus in patients submitted to fundoplication and Roux-en-Y distal gastrectomy.

The Barrx^TM^ system with TTS-1100 (15.7 mm length, 7.5 mm width) for focal ablation was used to treat smaller areas of metaplastic tissue. Different catheters were employed depending on the areas to be ablated (Figure 2).

### Parameters evaluation

Complications: frequency of early post-procedure complications.

Endoscopic images: comparison of Barrett´s epithelium length before and after the procedure.

Histology: eradication, residual presence, reappearance, or progression of IM/LGD.

### Definitions

Eradication: complete disappearance of goblet cells or LGD.

Residual island: presence of isolated islands of goblet cells or LGD after the procedure.

Reappearance: presence of anomalies is confirmed again after complete eradication of MI or LGD in the first or second control.

Progression: progression from IM to LGD or from LGD to HGD is confirmed

**Figure 3:**
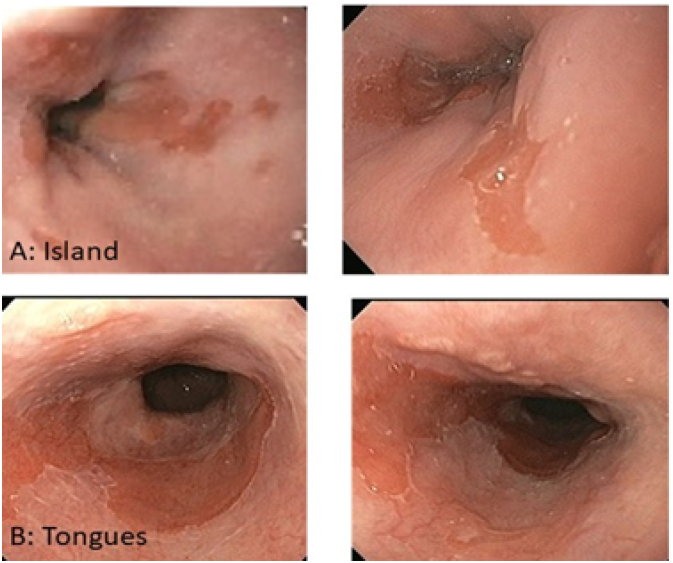
Residual islands (A) or tongues (B) of columnar epithelium after radiofrequency ablation.

Follow-up: patients were monitored by endoscopic and histologic analysis initially at the 6th month after the procedure. Biopsies were taken from both the residual columnar and squamous epithelium areas. Later, patients were evaluated annually with endoscopic and histological examination according to the same protocol. In APC group, the mean follow-up was 9.2 standard deviation ±1.9 years (range from 8 to 12 years) and in RFA group the mean follow-up was 7.6±2.1 years (range from 5 to 11 years). Depending on the endoscopic and histologic findings, patients were submitted to retreatment, mainly with APC, due to the presence of residual metaplastic islands or residual IM in Barrett's tongues. (Figure 3)

## RESULTS

The circumferential and maximal extensions of EGJ in the APC group were 30.47±8.05 mm and 54.76±9.81 mm, respectively. In patients undergoing RFA, the length of circumferential limit was 47.30±9.6 (p=0.07), and 71.33±18.1 mm for the maximal length (p=0.27) (Table 1).

After the initial endoscopic treatment, only one session of treatment with RFA was necessary to eliminate columnar epithelium in 26 of the 31 subjects (p=0.002), while seven patients (33.3%) needed two or three retreatment sessions after APC. In the RFA group, five patients had complementary retreatment: four were submitted to APC and one to ESD due to LGD (Table 2; Figure 3).

**Table 2:** Number of sessions required for complete initial eradication of endoscopic image of Barrett´s esophagus after argon plasma coagulation compared to radiofrequency ablation treatment.

	APC n=21	RFA n=31	p
Nº sessions (%)			
1	14 (66.6)	26 (83.9)	(p=0.02)
2	3 (14.3)	5 (16.1)*	
3	4 (19.1)		

APC: argon plasma coagulation; RFA: radiofrequency ablation; p: p-value.

* one patient submitted to endoscopic submucosal dissection, and four patients submitted to complementary argon plasma coagulation.

**Figure 4:**
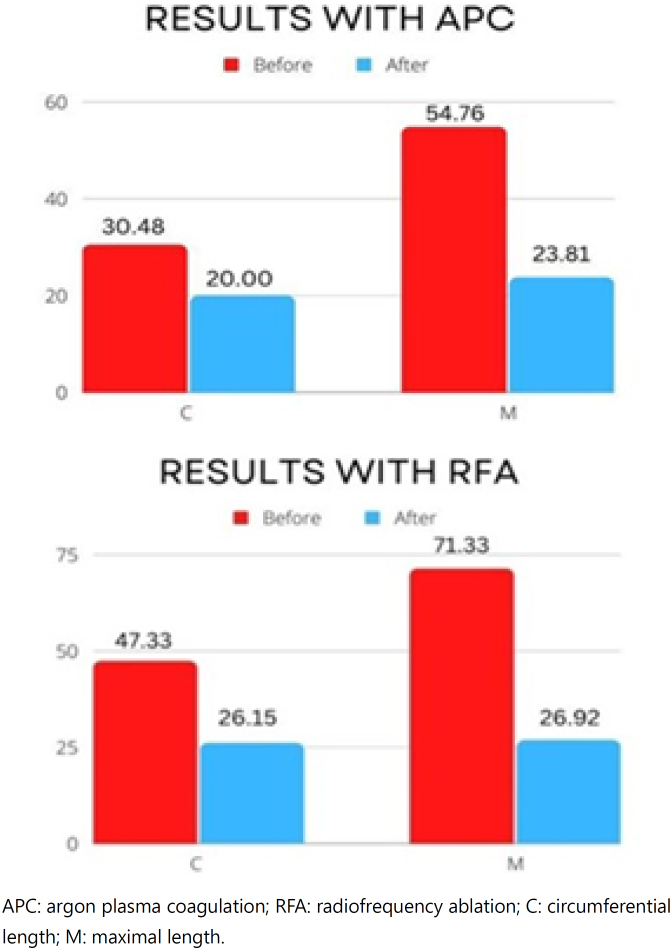
Circumferential and maximal length of Barrett´s epithelium before and after argon plasma coagulation and radiofrequency ablation in patients with Barrett´s esophagus submitted to fundoplication and Roux-en-Y distal gastrectomy.

The complications observed after both procedures are shown in Table 3. Strictures were successfully treated with dilatation with Savary's bougie. Table 4 shows the circumferential and maximal lengths of the metaplastic mucosa before and after each procedure. The decrease in circumferential length was 10.95mm in the group subjected to APC (p=0.09, p>0.05) and 21.15 mm in the group subjected to RFA. (p=0.0001, p<0.05). Regarding the decrease in maximal height after APC, it was 30.96 mm (p=0.0001, p<0.05) and 44.41 mm post RFA. (p=0.0001, p<0.05) (Figure 4). Table 5 shows the results of the histological study after endoscopic therapy. During the follow-up in the APC group, regression to carditis was observed in 14 patients, while 6 out of 20 patients presented residual island of IM or buried metaplastic cells that were subsequently subjected to retreatment with new sessions of APC.

**Table 3:** Complications observed after endoscopic argon plasma coagulation and radiofrequency ablation procedure in patients with Barrett´s esophagus.

	APC n=21 (%)	RFA n=31 (%)	p
Chest pain	18 (85.7)	31 (100)	0.06
Esophageal ulcer	2 (9.5)	3 (9.6)	1.00
Stricture	2 (9.5)*	1 (3.2)	0.55
Bleeding	0	0	
Perforation	0	0	
Mortality	0	0	

APC: argon plasma coagulation; RFA: radiofrequency ablation; p: p-value.

* endoscopic dilatation with Savary´s bougie.

**Table 4:** Extension of columnar epithelium area after the endoscopic treatment according Prague Classification^3^ before and after argon plasma coagulation and radiofrequency ablation (final results).

Prague Classification^3^		APC		RFA		p
		Before	After	Before	After	
C (mm)		30.47±8.05	20.00±7.75	47.30±9.6	26.15±5.2	
Difference (mm)		10.9		21.1		<0.016
M (mm)		54.76±9.81	23.80±7.40	71.33±18.1	26.92±4.8	
Difference (mm)		30.96		44.41		>0.18
	C3-5 M5	9	21	8	31	
	C5-7 M7	7	0	17	0	
	C>7 M7	5	0	6	0	

APC: argon plasma coagulation; RFA: radiofrequency ablation; C: circumferential length; M: maximal length; p: p-value.

**Table 5:** Histologic results after treatment with argon plasma coagulation or radiofrequency ablation in patients with intestinal metaplasia alone or with dysplasia: results observed during follow-up.

			APC n=21 (%)	RFA n=31 (%)	p
Intestinal metaplasia					
	Before treatment		20 (95.2)	25 (80.6)	<0.21
After treatment					
		Total initial eradication	14 (66.6)*	17 (54.8)*	<0.02
		Residual island	6 (28.6)†	8 (25.8)†	<0.02
		Reappearance during follow-up	1 (5)†	4 (12.9)†	>0.37
		Progression to LGD	0	0	
Intestinal metaplasia with LGD					
	Before treatment		1 (4.8)	6 (19.4)	>0.21
After treatment					
		Total initial eradication	1 (100%)*	4 (66.6%)*	>0.37
		Residual island of IM	0	2‡	1.0
		Reappearance IM during follow-up	1†	1†	>0.4
		Reappearance LGD during follow-up	0	0	
		Progression to HGD during follow-up	0	0	

APC: argon plasma coagulation; RFA: radiofrequency ablation; p-value; LGD: low-grade dysplasia; HGD: high-grade dysplasia.

* eradication with regression to carditis;

† treated with repeated APC;

‡ submitted to endoscopic submucosal dissection.

Complete eradication in one patient with IM and LGD was observed, but another patient also submitted to APC, presented reappearance of IM during follow-up. In the RFA group, regression to carditis with mucin-secreting cells was confirmed in 17 of 25 patients, but three patients showed residual IM (p=0.02, p<0.05), treated with complementary APC. In four out of six patients with IM with LGD, the complete eradication was achieved. In another patient, the appearance of LGD was observed at one-year follow-up and was submitted to ESD, while another patient presented reappearance of IM alone, being submitted to a new session of APC. The observed results demonstrated the efficacy of RFA in the treatment of these histological lesions.

Late complications from laparoscopic AS-DD operation before the endoscopic procedure were minimal. One patient with severe dysphagia, who required endoscopic dilation due to fundoplication stenosis, had no satisfactory outcome, so it was converted to Toupet partial fundoplication surgery, with no mortality. Another patient developed a hiatal hernia with “candy-cane syndrome” eight years later, and was submitted to revision surgery.

## DISCUSSION

BE is usually the result of severe chronic reflux disease. Reducing or eliminating esophageal epithelium's exposure to acid and bile is essential to achieve long-term regression of BE^13^. However, it is questionable whether the use of proton pump inhibitors or antireflux operations are more effective to accomplish this goal^10,31^.

Medical treatment of GERD proves ineffective at controlling LSBE^19,34^. Fundoplication can reduce both acid and bile reflux to normal levels, but patients with LSBE present more severe anatomical and pathophysiological alterations^33^.

Surgery in these cases cannot achieve an effective anti-reflux barrier. Post-surgical failure is observed three times more frequently than in non-Barrett's patients. (5–12% vs 12–39%)^24,25,50,51^. Therefore, the possibility of achieving regression of Barrett's metaplasia after fundoplication alone is rare and Barrett's progression to LGD and HGD as well as EAC following surgery is observed repeatedly^4,21^.

Due to the persistence of IM, efforts are focused on eliminating metaplastic mucosa and preventing disease progression, by combining endoscopic therapy with either medical or surgical treatment of GERD^27^. To achieve this objective, an effective treatment is required to eliminate acid and bile reflux. Some authors state repeated ablation sessions to obtain eradication, but these results are observed in non-operated patients, maintained only with medical treatment. Hence, some authors suggest performing antireflux surgery combined with eradication therapy in these patients^21,22^.

The combination of fundoplication plus an endoscopic APC or RFA procedure is published as a promising treatment for patients with BE and LGD at a follow-up of 6–8 years^20,40^. Some data suggest that individuals with prior fundoplication are more likely to have a durable response to eradication, although such an assertion remains inconclusive^40^.

RFA, performed either before or after surgery, is safe and effective for reducing or eliminating IM and dysplasia. The timing of the two procedures is debatable. For example, although fundoplication before ablation can straighten the esophagus and heal esophagitis, fundoplication itself can interfere with effective RFA by obscuring the EGJ landmarks or making access to the distal segments of the IM more difficult.

Performing surgery after ablation can also be more difficult due to transmural inflammatory changes, or complete ablation can never be possible because of an anatomical distortion of the esophagus (angulation, dilatation, and shortening)^6^. However, we performed the procedure with minimal difficulty. The results with RFA six months after surgery seem to be better than those observed with APC, with fewer complications and increased efficacy in eradicating IM and dysplasia, regardless of the number of sessions required. Eradication of the metaplastic epithelium is achieved in 59–62% of cases, with a percentage of buried cells between 19–52% after APC. The success in eradicating IM after RFA is 78–95%^20,42^. The most recent reports published these data up to 8–10 years of follow-up.

Our results on the modification of circumferential and maximal length of the metaplastic epithelium corroborate other studies which reporte a decrease in the BE length in −2.7 cm (range from −6 to −4 cm). Others observe a decrease in mean BE length from 6.2 to 1.2 cm after treatment (p=0.001). The higher number of RFA treatment (p<0.05) is associated with higher endoscopic and histologic success (p<0.05). All patients receiving three or more treatments have complete resolution of Barrett's metaplasia^21,37,46^.

A review of 18 studies, including 3,802 patients reported the efficacy of the treatment and six studies including 540 patients reported the durability. Complete eradication of MI was achieved in 78–92% (95%CI 70–86) of cases, complete eradication of dysplasia was achieved in 92% (95%CI 87–95), and progression to cancer was observed in 0.2–1.5% during treatment and 0.7% after eradication (95%CI 3–7)^1,2^.

Based on a recent meta-analysis, there is a significant reduction in the risk of progression to HGD or EAC among patients with BE-LGD treated with RFA compared to those undergoing endoscopic surveillance. Endoscopic eradication with RFA should be the preferred approach for BE-LGD^4,32,36^. However, the recurrence after initial complete eradication ranges from 21.5 to 33% of patients, but it does not assure that the acid and bile reflux is eliminated over time with medical treatment or fundoplication alone, which is obtained when AS-DD is performed. This could be considered the main advantage of our idea.

Adverse events are observed in about 20% (mainly pain) of cases and complications in less than 10%, the most frequent being esophageal stenosis in around 5–12%. Esophageal perforation is rare. Post-procedure mortality is not reported^23^. The results observed in the present study comparing APC and RFA are in accordance with other publications^2,30,34,43^.

The role of surgery is to provide an absolute barrier to acid and bile contents. But, in patients with LSBE, fundoplication alone can fail to control acid and bile reflux^1,8,48^. Therefore, we must perform AS-DD to avoid both types of refluxes (acid and bile), to obtain optimal results in terms of eradication and no progression of histological alterations in the long term. This technique has been performed by our group in LSBE patients for many years. In recent decades, it is recognized as a good alternative, especially in obese patients, reporting IM regression in more than 90% and dysplasia regression in 60% of cases, without progression to dysplasia or adenocarcinoma. The proposed technique for eradicating IM with or without LGD could offer even greater success^8,12,15,38^. However, there is only one case previously reported^43^, and so, that is the reason this option requires validation with further studies.

The limitations of this study are:

1. It is not a randomized study;
2. There is a low number of patients;
3. No manometric and 24-hr pH meter evaluations were performed; and
4. The follow-up is medium-term.

The strength of this study is that it provides results in a prospective cohort of patients with serial endoscopic and histologic evaluations.

## CONCLUSIONS

The elimination of metaplastic or dysplastic epithelium and the permanent reduction of acid and bile reflux are crucial to preventing BE progression. RFA combined with the AS-DD technique is feasible and can effectively treat BE lesions, avoiding progression to EAC, and probably could be the most suitable option without substantial morbidity and operative mortality.
